# Molecular Dynamics Investigations of Binding Mechanism for Triazoles Inhibitors to CYP51

**DOI:** 10.3389/fmolb.2020.586540

**Published:** 2020-09-25

**Authors:** Na Shi, Qingchuan Zheng, Hongxing Zhang

**Affiliations:** ^1^Laboratory of Theoretical and Computational Chemistry, Institute of Theoretical Chemistry, International Joint Research Laboratory of Nano-Micro Architecture Chemistry, College of Chemistry, Jilin University, Changchun, China; ^2^Key Laboratory for Molecular Enzymology and Engineering of the Ministry of Education, Jilin University, Changchun, China

**Keywords:** molecular dynamics simulations, MM-GB/SA, CYP51, triazoles, tunnels

## Abstract

The sterol 14α demethylase enzyme (CYP51) is an important target of fungal infections. However, the molecular mechanism between triazoles inhibitors and CYP51 remains obscure. In this study, we have investigated the binding mechanism and tunnel characteristic upon four triazoles inhibitors with CYP51 based on the molecular docking and molecular dynamics simulations. The results indicate the four inhibitors stabilize in the binding cavity of CYP51 in a similar binding mode. We discover a hydrophobic cavity (F58, Y64, Y118, L121, Y132, L376, S378, S506, S507, and M508) and the hydrophobic interaction is the main driving force for inhibitors binding to CYP51. The long-tailed inhibitors (posaconazole and itraconazole) have stronger binding affinities than short-tailed inhibitors (fluconazole and voriconazole) because long-tailed inhibitors can form more hydrophobic interactions with CYP51. The tunnel 2f is the predominant pathway for inhibitors ingress/egress protein, which is similar to the other works of CYP51. This study could provide the theoretical basis for the development of efficient azoles inhibitors and may lead a better insight into structure–function relationships of CYP51.

## Graphical Abstract

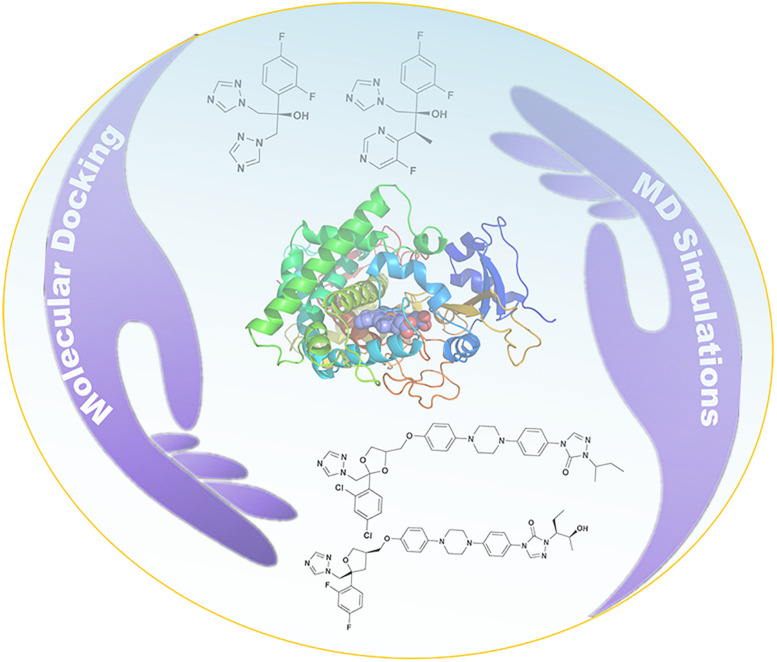

## Introduction

Life-threatening infections caused by fungi have increased rapidly, especially for patients that have immunocompromised diseases, such as AIDS, cancer, and organ-transplantation (Bongomin et al., 2017; Lee et al., 2020). It is reported that fungal diseases kill 1.5 million people per year, whose number is almost equal to the death of tuberculosis, and nearly three times as that of malaria (Brown et al., 2012; Bongomin et al., 2017). Among all kinds of fungal pathogens, *Candida albicans* is the most general fungi, which leads to candidemia (Antinori et al., 2016; Lee et al., 2020). To deal with the serious effects of life-threatening fungal infections, the development of antifungal agents has become a widespread concern (Lepesheva et al., 2018). In fungal cell membrane, the sterol 14α demethylase enzyme (CYP51) is responsible for catalyzing the lanosterol 14α methylation to produce ergosterol which can regulate the integrity, fluidity and permeability of the cell membrane (Balding et al., 2008; Lass-Floerl, 2011; Ene et al., 2012; Hargrove et al., 2016). Thus, influencing the growth and replication of fungi by inhibiting CYP51 has become a strategy for the development of antifungal agents (Lepesheva et al., 2008; Choi and Roush, 2017).

**FIGURE 1:**
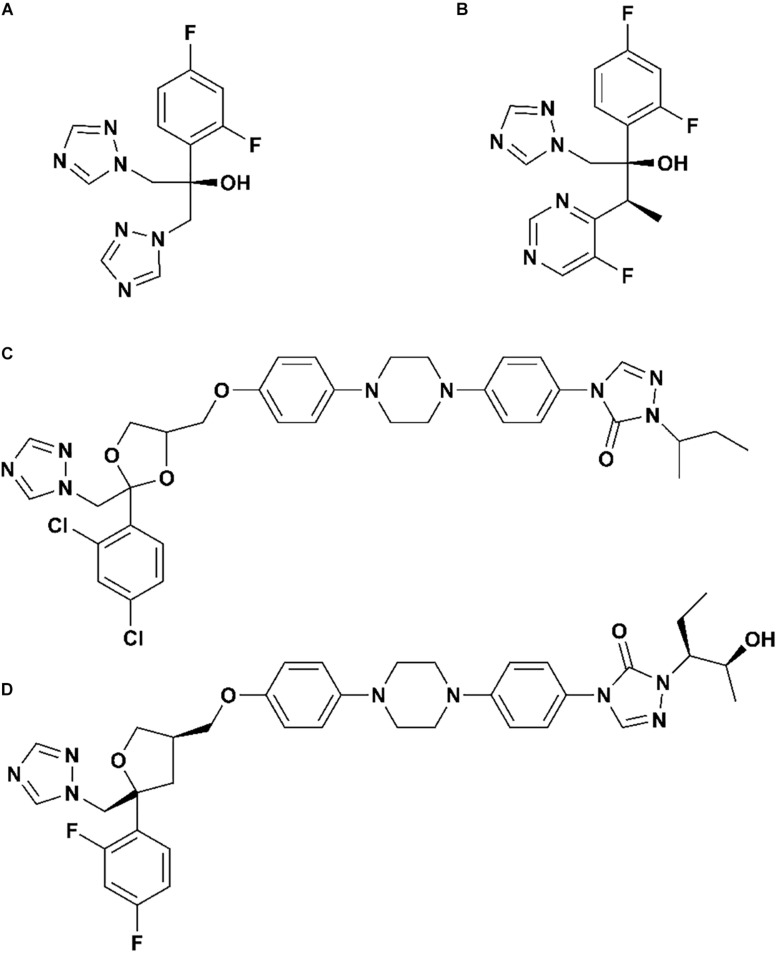
The structures of **(A)** fluconazole, **(B)** voriconazole, **(C)** itraconazole, and **(D)** posaconazole.

As we all know, there are four clinical triazoles agents targeting CYP51 for the treatment of systemic fungal infections: fluconazole (Flu), itraconazole (Itc), voriconazole (Vor), and posaconazole (Pos) (Lepesheva et al., 2018; Lee et al., 2020). The structures of four inhibitors are shown in Figure 1, they can be divided into short-tailed agents (ST: Flu and Vor) and long-tailed agents (LT: Itc and Pos) (Keniya et al., 2018). As the drug resistance mutations in CYP51 of *Candida albicans* increased, the effectiveness of four existing inhibitors is limited (Warrilow et al., 2019; Nishimoto et al., 2020). Therefore, elaborating the molecular mechanism of the existing drugs is very positive for further design and development of new drugs.

The successful resolution of the crystal structure of *Candida albicans* CYP51 by Lepesheva group inspired us to explore the molecular mechanisms between inhibitors and CYP51 (Hargrove et al., 2017). Molecular dynamics (MD) simulations are widely used to study the molecular mechanisms of inhibitors and enzyme (Watson et al., 2017; Sun et al., 2018; Xiao et al., 2020). Thus, we employed MD simulations and molecular docking to explore the binding mechanism between inhibitors and CYP51 in the present study. The results might offer insights into the structure–function relationships of CYP51 and provide the molecular basis for the rational design of new azoles inhibitors.

## Materials and Methods

### Preparation of Molecular Systems

The three-dimensional structure of *Candida albicans* CYP51 enzyme was obtained from the Protein Data Bank (PDB code: 5FSA) (Hargrove et al., 2017). Moreover, the structures of the four inhibitors: Flu (Compound CID: 3365), Vor (Compound CID: 71616), Itc (Compound CID: 55283), and Pos (Compound CID: 468595) were obtained from the PubChem database. After removing the ligand from the complex, the apo protein (Supplementary Figure S1) was saved by the Discovery Studio 3.1 (Studio, 2011). The CDOCKER protocol of Discovery Studio 3.1 (Studio, 2011) was employed to build the four complex structures, including Flu-CYP51 complex, Vor-CYP51 complex, Itc-CYP51 complex, and Pos-CYP51 complex.

### Molecular Docking

CDOCKER (Studio, 2011) is a grid-based molecular docking method by CHARMm force field. The geometry optimization of four ligands was performed by Gaussian 09 (Frisch et al., 2009) at the B3LYP/6-31G (d) level. The CHARMm force field was applied to ligands and receptors. The receptor was held rigid, whereas ligands were allowed to flex during the refinement. The input site sphere was defined as a radius of 12 and 18 Å for short-tailed inhibitors (Flu and Vor) and long-tailed inhibitors (Itc and Pos), respectively. The other docking information was set as the default value. A conformational search of the ligands was performed by a grid-based simulated annealing method. The ligands were firstly heated to 700 K (2000 steps) and then annealed to 300 K (5000 steps). The value of the grid extension was set as 8 Å. The top 20 poses of each complex were saved for comparison and analysis. Finally, combined with the information of binding site in the literature (Nair et al., 2016; Hargrove et al., 2017) and docking score, the bested conformation of each system was chosen as the initial structure for the subsequent MD simulations.

### Molecular Dynamics Simulations

For each system, geometry optimization of four inhibitors was performed by Gaussian 09 (Frisch et al., 2009) with the *ab initio* calculation method at the B3LYP/6-31G (d) level (Lee et al., 1988; Andersson and Uvdal, 2005). For charge derivation, the restrained electrostatic potential (RESP) fitting procedure was used (Bayly et al., 1993). The force field parameters of inhibitors were supplied by the general AMBER force field (GAFF) in the Antechamber module of AMBER 16 package (Wang et al., 2004, 2006; Case et al., 2016). The force field parameters developed by Shahrokh et al. were assigned to heme (Shahrokh et al., 2012). Finally, all missing atoms and hydrogen atoms were added using the t-leap procedure in the AMBER 16 package (Case et al., 2016). MD simulations were performed by the AMBER 16 package using the ff14SB force field (Maier et al., 2015; Case et al., 2016). To ensure the overall neutrality of the systems, counterions were added. All systems were subjected to MD simulations in explicit solvent, and all systems were solvated with TIP3P water box with 10 Å between the solute boundary (Yoo and Xantheas, 2011). First, protein and inhibitor were fixed with a 500 kcal/mol/Å^2^, and minimized the energy of all water molecules and counterions for 10000 steps of steepest descent (SD) followed by 10000 steps of the conjugate gradient. Subsequently, to remove conflicting contacts, the entire system was repeated for 12000 steps of SD minimization and 8000 steps of CG minimization. Next, the system was gradually heated up to 310 K in the NVT ensemble, thereby applying harmonic restraints with a force constant of 10.0 kcal/mol/Å^2^ on the solute atoms, and equilibration was performed three times with 3000 ps using a force constant of 5.0 kcal/mol/Å^2^ (Loncharich et al., 1992). Finally, 200 ns MD simulations were performed using the NPT ensemble without restraints. We used the Particle mesh Ewald (Darden et al., 1993) technique with a non-bonded cutoff of 12.0 Å to limit the direct space sum to treat the long-range electrostatic interactions. The SHAKE (Krautler et al., 2001) algorithm was used to constrain bonds involving hydrogen atoms. The time step of MD simulation was set to 2 fs, and sampling was performed every 10 ps into the MD file. Cluster analysis was performed by employing average linkage as the clustering algorithm (Shao et al., 2007). All figures in this contribution were generated by PyMOL (DeLano, 2002). The hydrogen bonds and hydrophobic interactions between the inhibitors and CYP51 were studied using LigPlot + v.2.2 (Laskowski and Swindells, 2011).

### Free Energy Calculations

The Molecular Mechanics Generalized Born Surface Area (MM-GB/SA) method implemented in AMBER 16 package was performed to calculate the binding free energy (Sun et al., 2014; Case et al., 2016), as well as the free energy decomposition of the four inhibitors systems. For each system, 2000 snapshots were selected from the last 100 ns MD trajectories to calculate the relevant energies. The binding free energy (Δ*G*_*bind*_) in MM-GB/SA between enzyme and ligand was summarized as the follows:

(1) $$\Delta \,G_{\mathrm{bind}}=G_{\mathrm{complex}}-\left(G_{\mathrm{receptor}}+G_{\mathrm{ligand}}\right)$$

(2) $$\Delta \,G=\Delta \,E_{\mathrm{MM}}+\Delta \,G_{\mathrm{sol}}-T\,\Delta \,S$$

(3) $$\Delta \,E_{\mathrm{MM}}=\Delta \,E_{\mathrm{int}}+\Delta \,E_{\mathrm{ele}}+\Delta \,E_{\mathrm{vdW}}$$

(4) $$\Delta \,G_{\mathrm{sol}}=\Delta \,G_{\mathrm{GB}}+\Delta \,G_{\mathrm{SA}}$$

In equation (2), Δ*E*_*MM*_, Δ*G*_*sol*_, and TS represent molecular mechanics components in the gas phase, the stabilization energy due to solvation, and a vibrational entropy term, respectively. Δ*E*_*MM*_ represents the summation of Δ*E*_*int*_, Δ*E*_*ele*_, and Δ*E*_*vdW*_ which are the internal, coulomb, and van der Waals interaction terms, respectively. Δ*G*_*sol*_ represents the solvation energy, which is divided into the electrostatic solvation free energy (Δ*G*_*GB*_) (Hawkins et al., 1996) and the non-polar solvation free energy (Δ*G*_*SA*_). Δ*G*_*GB*_ can be obtained by using the generalized Born method, and Δ*G*_*SA*_ is calculated as follows:

(5) $$\Delta \,G_{\mathrm{SA}}=\gamma \,\mathrm{SASA}+\beta$$

Here, γ and β, two empirical constants, were set as 0.0072 and 0.00 kcal/mol/Å^2^, respectively, and *SASA* (Weiser et al., 1999) represents the solvent accessible surface area determined by a probe radius of 1.4 Å. To estimate the change in conformational entropy (*T*Δ*S*) for all atoms, the normal-mode analysis was performed using the nmode module of AMBER 16 package (Case et al., 2016). 100 snapshots from the last 100 ns MD trajectories were used to calculate the entropic contribution.

Energy decompositions were performed to identify the important residues within the systems. Here, only per-residue decomposition was included, which was used to separate the energy contribution of each residue from the combination of enzyme with the inhibitor into three terms: van der Waals contribution (Δ*E*_*vdW*_), electrostatic contribution (Δ*E*_*ele*_), and solvation contribution (Δ*G*_*GB*_ + Δ*G*_*SA*_).

### Analysis of Access Tunnels

CAVER (Chovancova et al., 2012) is a famous software to explore routes leading from buried cavities (active sites) to enzyme surfaces. The starting point for the tunnel search was located in the position between heme and the inhibitor. The CAVER algorithm (Petrek et al., 2006) divides three-dimensional space into a grid and calculations are based on grid points. During calculations, the probe radius and the clustering threshold were set to 0.8 and 4.5 Å, respectively. A total of 200 frames of each system were extracted from the last 100 ns MD simulations trajectories. Other parameters were maintained at their default settings. Subsequently, tunnels were visualized by using PyMOL (DeLano, 2002).

## Results and Discussion

### Determination of the Optimal Binding Pose of Inhibitor by Docking Analysis

The CDOCKER protocol (Studio, 2011) is a CHARMm-based docking method, which was carried out to obtain an optimum initial model of the complex. To determine the reliability of this docking method, co-crystallized ligand (Pos) was firstly re-docked into defined cavity with the CDOCKER protocol. It has been reported that the distance between the nitrogen atoms on the triazole ring of ligands and the iron atom of heme (N6_*Flu*_-Fe, N1_*Vor*_-Fe, N4_*Pos*_-Fe; N7_*Itc*_-Fe) is less than 5 Å (Nair et al., 2016; Hargrove et al., 2017). The optimal conformation was the one with the best score among the structures that satisfy the above distance condition. The root-mean-square deviation (RMSD) value between the docking and initial conformation of Pos was 1.18 Å, which suggested that the CDOCKER protocol was suitable for docking in this work. The ligands Flu, Vor, and Itc were successively docked into the receptor, and the optimal binding pose was selected for the further MD analyses according to the above criteria. For the sake of clarity, Flu-CYP51 complex, Vor-CYP51 complex, Itc-CYP51 complex, and Pos-CYP51 complex was referred to as Flu system, Vor system, Itc system, and Pos system, respectively.”

### The Structural Stability and Dynamics Properties of the Inhibitor-CYP51 Systems

**FIGURE 2:**
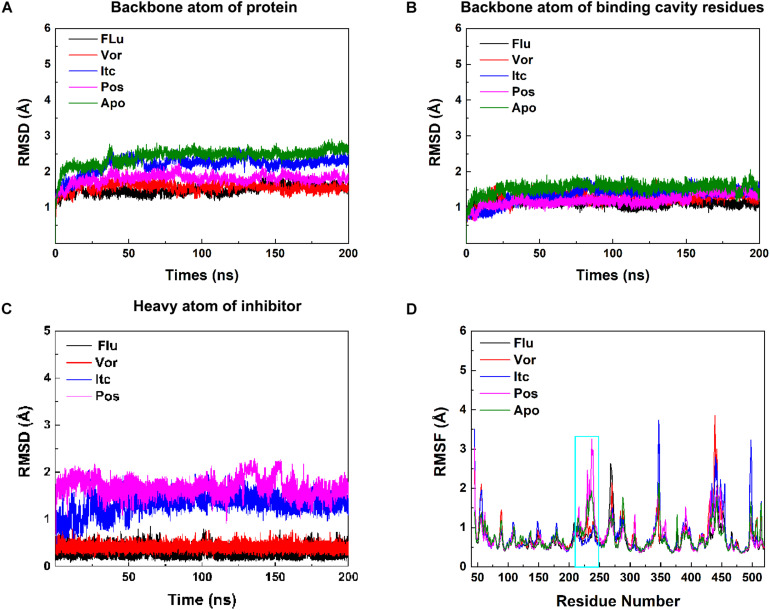
**(A)** RMSDs of backbone atoms of protein, **(B)** backbone atoms of binding cavity residues, and **(C)** heavy atoms of the inhibitor as a function of time. Black, red, blue, pink, and green for Flu, Vor, Itc, Pos, and Apo system, respectively. **(D)** RMSF values of the backbone Cα atom of each residue. The region of F-F′′ loop, F′′ helix, and F′′-G loop is shown in cyan rectangle.

In the 200 ns MD simulations of five systems, the root-mean-square deviation (RMSD) value of backbones atom of protein, binding cavity residues, and heavy atom of inhibitors were calculated to investigate the structural stability of CYP51. As shown in Figures 2A–C, each system gradually reached equilibrium, which remained quite stable during the last 100 ns. Thus, all subsequent analysis was performed on the last 100 ns of the simulation trajectories. Comprehensively considered these RMSD values of systems, the binding of the inhibitors reduced the perturbation of the protein to some extent. To further explore the effect of inhibitors binding on fluctuations of a certain residue, the root-mean-square-fluctuation (RMSF) of backbones Cα atoms in CYP51 was calculated (Figure 2D). The comparison of RMSF between the Apo system and four inhibitors systems showed that the fluctuations were mostly similar except for the structural elements of F-F′′ loop, F′′ helix, and F′′-G loop (Supplementary Figure S1). This region is also called F-G loop in P450 enzyme, which may affect the channel characteristics of the enzyme (Cojocaru et al., 2007). The F- F′′ loop, F′′ helix, and F′′-G loop showed large RMSF values in Apo system (Figure 2D). Compared with Apo system, the RMSF values of Flu, Vor, and Itc systems were reduced, while that of Pos system was increased. Further analysis showed that due to the 2-hydroxypentan of Pos is close to the F-G loop, the instability of 2-hydroxypentan (Supplementary Figure S2) caused the F-G loop to change greatly, thus increasing the RMSF value of the F-G loop. These results indicated that inhibitors binding may affect protein tunnel characteristics by influencing the conformations of F- F′′ loop, F′′ helix, and F′′-G loop. These results suggested that inhibitors’ binding increased the stability of CYP51. Local conformational changes of F-G loop caused by inhibitors binding may affect the protein tunnel characteristics.

### Analysis Inhibitor Binding Mode

**FIGURE 3:**
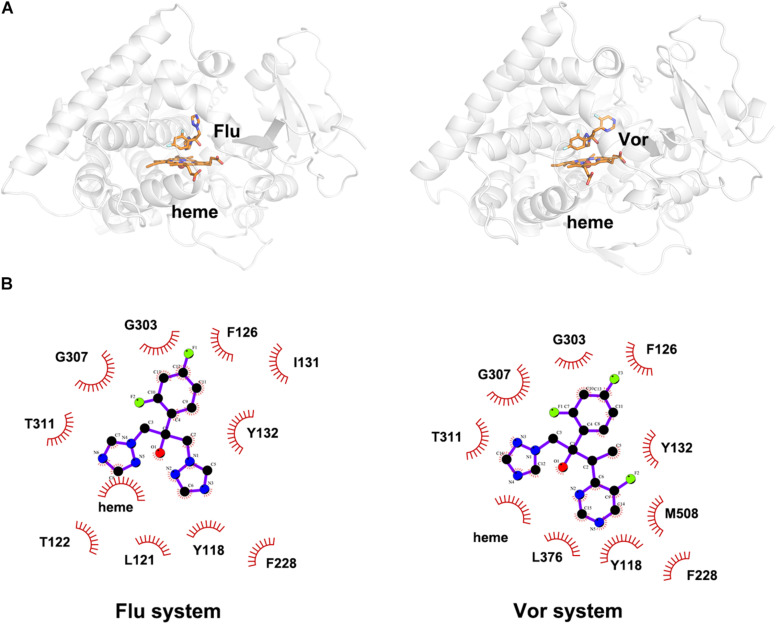
**(A)** The representative structures of Flu system and Vor system. **(B)** The 2D diagrams of the detailed binding information of Flu system and Vor system. The protein is shown in white cartoon, the inhibitor and heme are displayed in orange sticks. The molecular interactions show hydrophobic interactions as semi-arcs with red eyelashes.

**FIGURE 4:**
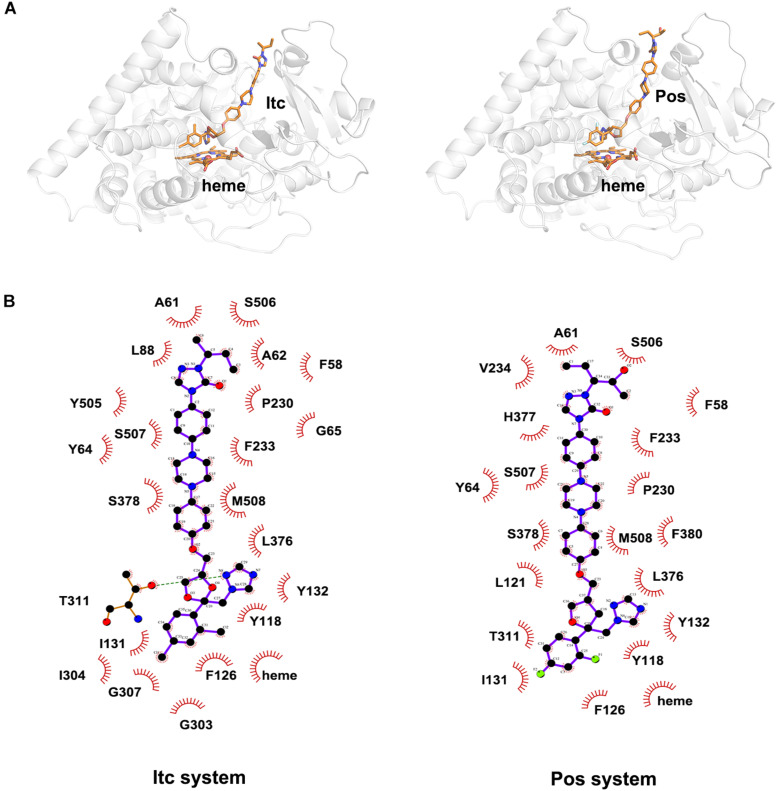
**(A)** The representative structures of Itc system and Pos system. **(B)** The 2D diagrams of the detailed binding information of Itc system and Pos system. The protein is shown in white cartoon, the inhibitor and heme are displayed in orange sticks. The molecular interactions show hydrogen bonds as green dashed lines and hydrophobic interactions as semi-arcs with red eyelashes.

To explore the binding mode of inhibitor in the binding cavity of CYP51, clustering analysis was used to extract the representative conformation. As shown in Figures 3A, 4A, the shared triazole ring of four inhibitors located above the heme was coordinated with the heme. The halogenated phenyl was pointed toward to the crack between the I helix and the B′-C loop. The rest side chain oriented toward the entrance of the binding cavity of CYP51. These results indicated the four inhibitors maintained a similar binding pattern. The 2D diagrams displayed the interaction between inhibitor and protein. As shown in Figures 3B, 4B, the hydrophobic interaction was the main driving force for inhibitors binding to CYP51. Based on molecular shapes and scaffolds, the four inhibitors were divided into two kinds of inhibitors: ST inhibitors and LT inhibitors (Keniya et al., 2018). ST inhibitors (Flu and Vor) formed hydrophobic interaction with the shared residues Y118, F126, Y132, F228, G303, G307, and T311 (Figure 3B). For LT inhibitors (Itc and Pos), they formed hydrophobic interaction with the common residues F58, Y64, Y118, L121, Y132, L376, S378, S506, S507, and M508 (Figure 4B). The important and obvious discrepancy of two kinds of inhibitors was the length of the side chain. Comparing with ST inhibitors, the extended side chains of LT inhibitors (Itc and Pos) provided additional points in contact with the azole target CYP51. Thus, LT inhibitors can form more hydrophobic interactions with CYP51 than ST inhibitors, which may demonstrate LT inhibitors have stronger binding affinities with CYP51.

The analysis of inhibitor binding mode suggested four inhibitors hold a similar binding pattern and the hydrophobic interactions were the dominant driving force for inhibitors’ binding to CYP51. For both types of inhibitors, LT inhibitors can form hydrophobic interactions with more residues due to the characteristics of their long side chains. This may indicate that LT types of inhibitors are more suitable for targeted CYP51.

### Rational Ranking of Binding Ability by Binding Free Energy Calculations

To gain energic information about the four inhibitors systems, the binding free energy calculations were performed by the MM-GB/SA method, and the entropy contributions were also considered. As presented in Table 1, the total Δ*G*_*bind*_ values of Flu, Vor, Itc, and Pos systems were −8.50, −15.08, −52.52, and −48.53 kcal/mol, respectively. LT inhibitors (Pos and Itc) had stronger binding affinities when compared with ST inhibitors (Flu and Vor), which confirmed our previous speculation. Our results showed the rational ordering of binding affinities in different systems which were consistent with that of the experimental inhibitory effects (Hargrove et al., 2017). Further analysis of the binding free energy indicated that the contributions of van der Waals interactions (Δ*E*_*vdW*_), electrostatic energy (Δ*E*_*ele*_), and non-polar solvation energy (Δ*G*_*non–polar*_) were favorable for the formation of the inhibitors’ complexes. As listed in Table 1, non-polar interaction (Δ*G*_*non–polar*_) was mainly responsible for the formation of the Flu system (−47.97 kcal/mol), Vor system (−54.83 kcal/mol), Pos system (−101.34 kcal/mol), and Itc system (−100.09 kcal/mol). In comparison to the non-polar interaction, polar interaction of the four systems had an unfavorable contribution. The entropy change values of the four systems were less than zero, which were detrimental to the binding of the inhibitors to the CYP51. The results of free energy analysis elaborated that non-polar interaction was the key factor for the binding of inhibitors and CYP51.

**TABLE 1:** Binding free energies (kcal/mol) and its components of four inhibitors systems.

	**Flu**	**Vor**	**Itc**	**Pos**
Δ*E*_*vdW*_	–42.98	–49.12	–90.33	–91.32
Δ*E*_*ele*_	–6.48	–1.31	–7.00	–12.81
Δ*G*_*GB*_	25.78	21.62	23.53	35.44
Δ*G*_*SA*_	–4.99	–5.71	–9.76	–10.02
Δ*E*_*polar*_^*a*^	19.30	20.31	16.53	22.63
Δ*E*_*non–polar*_^*b*^	–47.97	–54.83	–100.09	–101.34
Δ*G*_*mmgbsa*_^*c*^	–28.67	–34.51	–83.56	–78.71
*T*Δ*S*	–20.17	–19.43	–31.04	–30.18
Δ*G*_*bind*_^*d*^	–8.50	–15.08	–52.52	–48.53

*^*a*^Δ*E*_polar_ = Δ*E*_ele_ + Δ*G*_*GB*_. ^*b*^Δ*E*_*non–polar*_ = Δ*E*_vdW_ + Δ*G*_*SA*_. ^*c*^Δ*G*_mmgbsa_ = Δ*E*_vdW_ + Δ*E*_ele_ + Δ*G*_GB_ + Δ*G*_*SA*_. ^*d*^Δ*G*_bind_=Δ*G*_*mmgbsa*_ – *T*Δ*S*.*

The total binding free energy was decomposed into residues to identify key residues for inhibitors binding to CYP51. Essential residues with the binding free energy value below −1.0 kcal/mol were listed in Table 2. The number of residues meeting to the criterion were 4, 3, 14, and 12 in four systems, respectively, which also indicated that the LT inhibitors were tightly bound to CYP51. We found that Y118 and L376 had significant contributions in inhibitors binding of all four inhibitors systems, Y64, L87, L88, P230, F233, F380, and M508 made outstanding contributions during the LT inhibitors binding.

**TABLE 2:** Decomposition of binding free energy (kcal/mol) on per residue basis for Flu system, Vor system, Pos system, and Itc system.

**Residue**	**Flu**	**Vor**	**Itc**	**Pos**
Y118	–1.84	–2.90	–1.82	–2.03
F126	–0.92	–0.53	–1.05	–0.71
Y132	–1.39	–0.82	–0.71	–0.85
F228	–1.02	–0.74	–0.88	–0.24
T311	–0.27	–0.99	–2.78	–0.98
L376	–1.91	–2.19	–1.90	–2.11
M508	–0.76	–2.40	–3.63	–3.41
F380	–0.23	–0.74	–1.02	–1.09
A61	0.01	0.01	–0.70	–1.37
Y64	0.01	–0.02	–1.07	–1.40
L87	0.01	0.04	–1.19	–1.08
L88	0.01	0.02	–1.08	–1.33
P230	0.01	–0.02	–2.38	–1.25
F233	–0.06	–0.24	–1.04	–1.47
H310	–0.74	–0.62	–2.16	–0.54
S506	0.02	0.06	–1.21	–1.36
S507	–0.05	–0.07	–1.00	–1.01

### Tunnel Analysis

**FIGURE 5:**
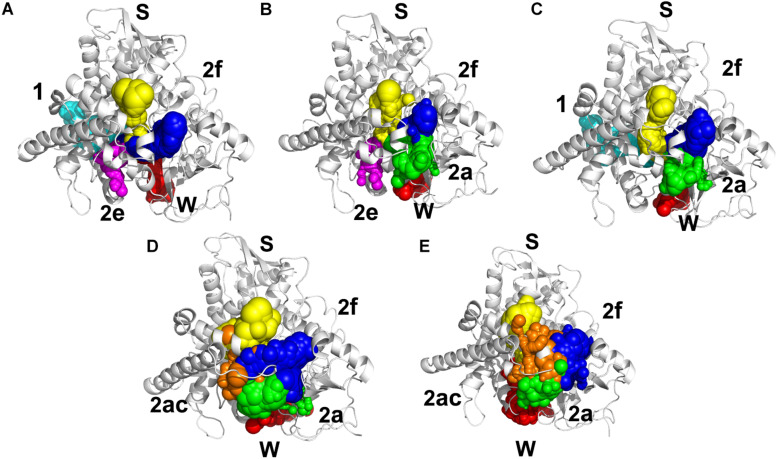
Access tunnel identified from the average structures of **(A)** Flu system, **(B)** Vor system, **(C)** Itc system, **(D)** Pos system, and **(E)** Apo system, respectively. The protein backbone is shown in cartoon. Tunnels are shown in sphere for blue (2f), green (2a), magenta (2e), orange (2ac), yellow (s), red (w), and cyan (1).

Illustrating the tunnel characteristic of CYP51 is beneficial to develop new inhibitors and understand the structure–function relationships of CYP51 (Yu et al., 2016; Fischer et al., 2018). In this work, 200 frames were extracted from the last 100 ns trajectories to classify and analyze the characteristics of access pathways in four inhibitors systems and Apo system. The tunnels were clustered by the average-link algorithm according to the pairwise distances of tunnels. On the basis of spatial and secondary structure, the nomenclature of these tunnels is defined systematically by Wade group (Cojocaru et al., 2007). The five highest ranked tunnels of five systems were all displayed in Figure 5, and the characteristics of these tunnels were summarized in Table 3. As shown in Figure 5, the locations of five tunnels were marked with different color spheres (Flu system: 2f, W, S, 1, and 2e; Vor system: 2f, 2a, W, S, and 2e; Itc system: 2f, S, W, 1, and 2a; Pos system: 2f, 2a, W, S, and 2ac; Apo system: 2f, S, W, 2a, and 2ac). Tunnels 2f, 2a, 2e, and 2ac are subclasses of tunnel 2. Tunnel 2f locates between the F-G loop, Pro-rich loop, and A helix, whereas tunnel 2a locates between the F-G loop and B′-C loop (Supplementary Figure S1). Tunnel 2e egresses through the B′-C loop, and tunnel 2ac egresses between the B′ helix and the G helix (Supplementary Figure S1). Tunnel W (water tunnel) egresses at the base of the B′-C loop near the C-terminus of the B helix, and tunnel S (solvent tunnel) runs between the E, F, and I helices and β5 sheet (Supplementary Figure S1). As listed in Table 3, tunnel 2f was the most frequently identified collective pathway and had the highest bottlenecks radius in five systems. Thus, tunnel 2f was regarded as the predominant tunnel for inhibitors ingress/egress from the active site to the surface of CYP51, which was similar as the other works of CYP51 (Monk et al., 2014; Yu et al., 2016; Gao et al., 2018). The occurrence of tunnel 2f was different slightly in five systems, which may be related to the size of the inhibitors. In the LT systems (Itc and Pos), the inhibitor is long and bulky, and its binding mode analysis showed that its long side chain extended to the entrance of the tunnel 2f, resulting in a fully opened tunnel 2f. In the ST systems (Flu and Vor), the inhibitor is in small size, and the inhibitor was submerged in the binding cavity of CYP51, causing decreased slightly in the opening frequency of tunnel 2f, which was in line with the RMSF analysis that inhibitors’ binding affected the tunnel characteristic. Further, we determined the essential residues lining the dominant tunnel 2f, and all residues located within the 3 Å distance from the tunnel surface will be regarded as tunnel-lining residues (Supplementary Table S1). The key residues (F58, Y64, Y118, L121, Y132, L376, S378, S506, S507, and M508) determined based on the binding mode analysis and per-residue binding free energy decomposition analysis also belong to the tunnel-lining residues. Most of the tunnel-lining residues were hydrophobic residues, which formed a stable hydrophobic cavity and provided hydrophobic interactions that play an indispensable role in inhibitor stabilization. Thus, when designing more efficient inhibitors, the interactions between inhibitors and these residues should be rationally increased and the new inhibitors should be hydrophobic ligands.

**TABLE 3:** Characteristics of the five top ranked tunnels of Flu system (A), Vor system (B), Itc system (C), Pos system (D), and Apo system, respectively.

**(A) Characteristics of Flu system**					
**Tunnel**	**2f**	**W**	**S**	**1**	**2e**
Occurrence	93%	82%	34%	25%	11%
Mean bottleneck radius [Å]	1.26	0.99	0.96	0.91	0.84
Max. bottleneck radius [Å]	1.74	1.26	1.45	1.19	0.97
Mean pathway length [Å]	23.86	23.50	21.14	29.70	21.40
**(B) Characteristics of Vor system**					
**Tunnel**	**2f**	**2a**	**W**	**S**	**2e**

Occurrence	98%	84%	77%	27%	15%
Mean bottleneck radius [Å]	1.26	1.08	0.92	0.97	0.83
Max. bottleneck radius [Å]	2.08	1.83	1.18	1.47	0.93
Mean pathway length [Å]	25.88	28.70	24.08	21.80	2
**(C) Characteristics of Itc system**					
**Tunnel**	**2f**	**S**	**W**	**1**	**2a**

Occurrence	100%	89%	85%	51%	34%
Mean bottleneck radius [Å]	2.00	1.09	0.95	0.93	0.93
Max. bottleneck radius [Å]	2.46	1.56	1.30	1.31	1.34
Mean pathway length [Å]	16.95	20.23	23.69	33.58	20.95
**(D) Characteristics of Pos system**					
**Tunnel**	**2f**	**2a**	**W**	**S**	**2ac**

Occurrence	100%	86%	81%	38%	16%
Mean bottleneck radius [Å]	1.86	1.78	0.93	1.03	1.00
Max. bottleneck radius [Å]	2.25	2.39	1.23	1.89	1.69
Mean pathway length [Å]	24.65	24.20	22.24	19.56	24.60
**(E) Characteristics of Apo system**					
**Tunnel**	**2f**	**S**	**W**	**2a**	**2ac**

Occurrence	88%	80%	69%	65%	38%
Mean bottleneck radius [Å]	1.69	1.34	0.91	1.44	1.40
Max. bottleneck radius [Å]	2.37	1.72	1.40	2.03	2.10
Mean pathway length [Å]	20.92	19.93	23.31	22.53	22.82

## Conclusion

The sterol 14α-demethylase enzyme (CYP51) belongs to cytochrome P450 family essential in sterol biosynthesis, which is the target for fungal infections. In this work, molecular docking and molecular dynamics simulations were employed to investigate the binding mechanism and tunneling characteristics between four inhibitors and CYP51, so as to provide useful information for inhibitors design. The results show that four inhibitors bind CYP51 in similar binding mode and hydrophobic interactions are the main driving force for inhibitors binding to CYP51. Due to long-tailed inhibitors (posaconazole and itraconazole) can contact with more residues of CYP51 by hydrophobic interactions than short-tailed inhibitors (fluconazole, voriconazole), long-tailed inhibitors have stronger binding affinities. Tunnel analysis showed that tunnel 2f is the predominant pathway for inhibitors ingress/egress from the active site to the surface of CYP51. We discover a hydrophobic cavity and identify the key residues (F58, Y64, Y118, L121, Y132, L376, S378, S506, S507, and M508) which are responsible for anchoring the inhibitors binding to CYP51. Therefore, in order to enhance the binding affinity of inhibitors to CYP51, we should focus on strengthening hydrophobic interactions of inhibitors and these residues, while longer inhibitors are probably best suited to target CYP51. Taken together, the results obtained in this study will be valuable for designing potent azoles inhibitors and improve the understanding of the structure–function relationships of CYP51.

## Data Availability Statement

The datasets presented in this study can be found in online repositories. The names of the repository/repositories and accession number(s) can be found in the article/ Supplementary Material.

## Author Contributions

NS performed the research, analyzed the data, and wrote the manuscript. QZ and HZ designed the research and revised the manuscript. All authors contributed to the article and approved the submitted version.

## Conflict of Interest

The authors declare that the research was conducted in the absence of any commercial or financial relationships that could be construed as a potential conflict of interest.
