# Supplementary material for: Molecular Dynamics Investigations of Binding Mechanism for Triazoles Inhibitors to CYP51
Source: Front Mol Biosci. 2020 Sep 25;7:586540. doi: 10.3389/fmolb.2020.586540 (PMC7546855; doi:10.3389/fmolb.2020.586540)
Supplement: Supplementary file 1 [file Data_Sheet_1.docx]

Supplementary Material

## Supplementary Figures and Tables


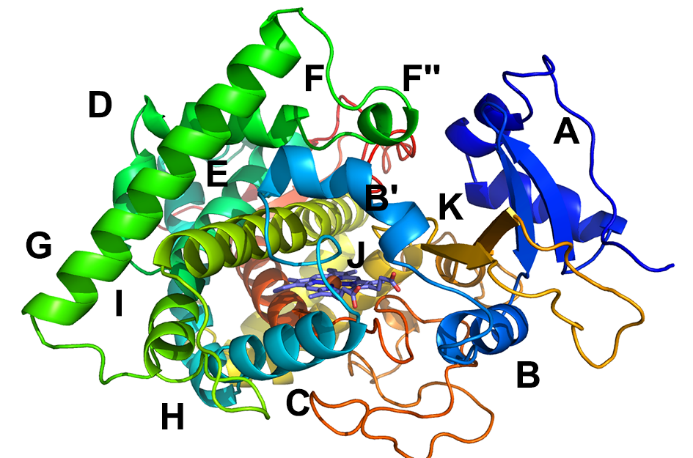


**Figure** **S1.** The overall structure of CYP51, colored from N-terminal (blue) to C terminal (red).





Figure S2. RMSF values of the heavy atom of Pos.

**Table S1.** Occurrences (>50%) of the bottleneck residues of tunnel 2f in four inhibitors systems.

| **Residue** | **Occurrence** | | | |
| --- | --- | --- | --- | --- |
|  | **Flu** | **Vor** | **Itc** | **Pos** |
| F58 | 42% | 74% | 51% | 77% |
| A61 | 91% | 97% | 97% | 90& |
| A62 | 58% | 80% | 68% | 61% |
| Y64 | 93% | 97% | 100% | 96% |
| G65 | 93% | 96% | 98% | 89% |
| Q66 | 69% | 64% | 60% | 32% |
| L87 | 93% | 98% | 100% | 100% |
| L88 | 91% | 98% | 92% | 100% |
| M92 | 25% | 38% | 51% | 50% |
| A117 | 9% | 43% | 51% | 86% |
| Y118 | 93% | 98% | 100% | 100% |
| L121 | 90% | 96% | 100% | NA |
| T122 | 93% | 98% | 98% | 100% |
| F126 | 93% | 98% | 89% | 100% |
| I131 | 93% | 98% | 55% | 100% |
| Y132 | 93% | 98% | 94% | 100% |
| F228 | 93% | 98% | 100% | 95% |
| T229 | 30% | 44% | 76% | 21% |
| P230 | 93% | 98% | 100% | 96% |
| I231 | 44% | 29% | 93% | 68% |
| F233 | 77% | 88% | 98% | 73% |
| V234 | NA | NA | NA | 96% |
| M306 | 93% | 98% | 14% | 100% |
| G307 | 93% | 98% | 79% | 100% |
| G308 | 93% | 98% | NA | 100% |
| H310 | 87% | 85% | 55% | 98% |
| T311 | 93% | 98% | 68% | 100% |
| P375 | 58% | 18% | NA | NA |
| L376 | 93% | 98% | 100% | 100% |
| H377 | 93% | 93% | 100% | 97% |
| S378 | 93% | 95% | 100% | 100% |
| I379 | 93% | 97% | 100% | 99% |
| F380 | 93% | 98% | 100% | 100% |
| R381 | 46% | 79% | 45% | 37% |
| L403 | NA | NA | 61% | 23% |
| Y505 | 92% | 96% | 96% | 93% |
| S506 | 88% | 97% | 70% | 89% |
| S507 | 89% | 96% | 99% | 100% |
| M508 | 93% | 98% | 100% | NA |
| V509 | 91% | 83% | 99% | 95% |

NA: the residue not detected in tunnel 2f.
